# The first complete chloroplast genome sequence from *Polemonium chinense* (Polemoniaceae)

**DOI:** 10.1080/23802359.2019.1668734

**Published:** 2019-09-25

**Authors:** Jianfang Li, Qian Yang, Bei Xu, Zhan-Lin Liu

**Affiliations:** Key Laboratory of Resource Biology and Biotechnology in Western China (Ministry of Education), College of Life Sciences, Northwest University, Xi'an, China

**Keywords:** *Polemonium chinense*, Polemoniaceae, chloroplast genome, phylogeny

## Abstract

In this study, the complete chloroplast genome of *Polemonium chinense* was determined by next-generation sequencing technology. The plastome is 155,578 bp in length, with a large single-copy region (LSC) of 85,864 bp and a small single-copy region (SSC) of 18,246 bp, separated by a pair of inverted repeat regions (IRs) of 25,734 bp. It contains 135 genes, including 89 protein-coding genes, 37 tRNA genes, 8 rRNA genes, and 1 pseudogene. The overall GC content is 37.1%, while the corresponding values in the LSC, SSC and IR region are 35.1, 30.3, and 42.9%, respectively. The phylogenetic tree shows that Polemoniaceae is sister to Fouquieriaceae.

Polemoniaceae, the phlox family (Ericales), includes 270–400 species of annual and perennial herbs, rarely subshrubs or vines, with the diversity center in western North America. The family is taxonomically complex, its phylogenetic position has been controversial for many years. Clade of Polemoniaceae + Fouquieriaceae was supported by molecular data (Rose et al. 2018; Li et al. 2019) but objected by Savolainen et al. (2000). Yan et al. (2018) found that Polemoniaceae was clustered to Primulaceae with weak support values. At a low taxonomical level, generic and species delimitation within Polemoniaceae has been still unsolved. According to the Plant List, Polemoniaceae includes 29 genera while only 18 genera are accepted by APG IV (Angiosperm Phylogeny Group 2016). Recently, together with the rapid development of the next-generation sequencing technologies, chloroplast genome has widely been used for phylogeny reconstruction due to its conserved structure and plenty of information (Li et al. 2019). In this study, we determined the complete chloroplast (cp) genome of *Polemonium chinense*, which is expected to provide new data for classification and phylogenetic study of Polemoniaceae.

The total genomic DNA was extracted from fresh leaves of *Polemonium chinense* collected in Qinling Mountains, China (N33.54° E108.55°). The voucher (2016LIU332) was deposited at the Evolutionary Botany Laboratory (EBL), Northwest University. Data processing followed the previous method (Peng et al. 2019) including sequence trimming, assembling and gene annotations. The genome was annotated manually with *Hydrocera triflora* (NC037400) as a reference.

The whole chloroplast genome of *Polemonium chinense* (GenBank accession number MN057953) is 155,578 bp in length, with a large single-copy region (LSC) of 85,864 bp and a small single-copy region (SSC) of 18,246 bp, separated by a pair of inverted repeat regions (IRs) of 25,734 bp. It contains 135 genes, including 89 protein-coding genes, 37 tRNA genes, eight rRNA genes and one pseudogene (*rps19*). Seventeen genes are duplicated in the IRs, containing six protein-coding genes (*rpl2*, *rpl23*, *ycf2*, *ycf15*, *ndhB* and *rps7*), seven tRNA genes (*trnI-CAU*, *trnL-CAA*, *trnV-GAC*, *trnI-GAU*, *trnA-UGC*, *trnR-ACG*, *trnN-GUU*) and four rRNA genes (*rrn16*, *rrn23*, *rrn4.5*, *rrn5*). Among the annotated genes, 13 (*rps16*, *atpF*, *rpoC1*, *rps12*, *petB*, *rpl2*, *ndhB*, *ndhA*, *trnK-UUU*, *trnL-UAA*, *trnV-UAC*, *trnI-GAU*, *trnA-UGC*) contain a single intron and two genes (*ycf3* and *clpP*) include two introns. The overall GC content is 37.1%, while the corresponding values in the LSC, SSC, and IR region are 35.1, 30.3, and 42.9%, respectively. The protein-coding genes of 32 complete chloroplast genomes in Ericales were used to construct the phylogenetic tree by the maximum likelihood method (Peng et al. 2019), with *Curtisia dentata* (MG524999) and *Cornus controversa* (MG525004) as an outgroup. The phylogenetic tree supports that Polemoniaceae is sister to Fouquieriaceae (Figure 1). The plastome data of *Polemonium chinense* will provide new insight into the evolutionary progress of Ericales and help to identify the phylogenetic relationships within Polemoniaceae.

**Figure 1.**
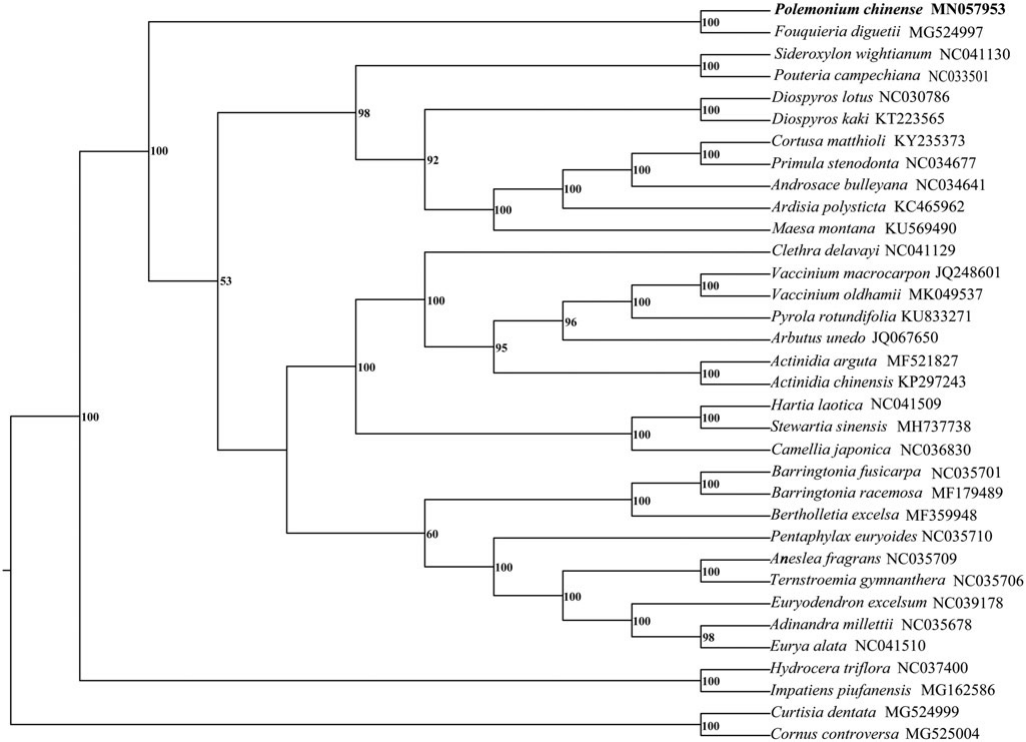
The phylogenetic tree of 34 representatives based on protein-coding genes of the complete chloroplast genomes. Bootstrap values are labeled beside the branches.
